# Cross‐Modality Comparison of Fetal Brain Phenotypes: Insights From Short‐Interval Second‐Trimester MRI and Ultrasound Imaging

**DOI:** 10.1002/hbm.70349

**Published:** 2025-10-01

**Authors:** Madeleine K. Wyburd, Nicola K. Dinsdale, Vanessa Kyriakopoulou, Lorenzo Venturini, Robert Wright, Alena Uus, Jacqueline Matthew, Emily Skelton, Lilla Zöllei, Joseph Hajnal, Ana I. L. Namburete

**Affiliations:** ^1^ Oxford Machine Learning in NeuroImaging Lab, Department of Computer Science University of Oxford Oxford UK; ^2^ Research Department of Imaging Physics and Engineering, School of Biomedical Engineering and Imaging Sciences King's College London London UK; ^3^ Research Department of Early Life Imaging, School of Biomedical Engineering and Imaging Sciences King's College London London UK; ^4^ Fetal Medicine Department GSTT London UK; ^5^ School of Health and Psychological Sciences City, University of London London UK; ^6^ Athinoula A. Martinos Center for Biomedical Imaging, Department of Radiology Massachusetts General Hospital Boston Massachusetts USA; ^7^ Harvard Medical School Charlestown Massachusetts USA

## Abstract

Advances in fetal three‐dimensional (3D) ultrasound (US) and magnetic resonance imaging (MRI) have revolutionized the study of fetal brain development, enabling detailed analysis of brain structures and growth. Despite their complementary capabilities, these modalities capture fundamentally different physical signals, potentially leading to systematic differences in image‐derived phenotypes (IDPs). Here, we evaluate the agreement of IDPs between US and MRI by comparing the volumes of eight brain structures from 90 subjects derived using deep‐learning algorithms from majority same‐day imaging (days between scans: mean = 1.2, mode = 0 and max = 4). Excellent agreement (intra‐class correlation coefficient, ICC>0.75) was observed for the cerebellum, cavum septum pellucidum, thalamus, white matter and deep grey matter volumes, with significant correlations $\left(p<0.001\right)$ for most structures, except the ventricular system. Bland–Altman analysis revealed some systematic biases: intracranial and cortical plate volumes were larger on US than MRI, by an average of $35\;{\mathrm{cm}}^{3}$ and $4.1\;{\mathrm{cm}}^{3}$, respectively. Finally, we found the labels of the brainstem and ventricular system were not comparable between the modalities. These findings highlight the necessity of structure‐specific adjustments when interpreting fetal brain IPDs across modalities and underscore the complementary roles of US and MRI in advancing fetal neuroimaging.

## Graphical abstract

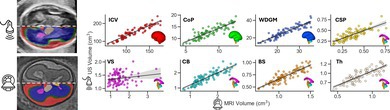

## 1 Introduction

The measurement of individual brain structures provides valuable insights into fetal development, with deviations from normative measures often associated with *at‐risk* pregnancies (Polat et al. 2017; Sadhwani et al. 2022). In routine prenatal care, fetal development is typically monitored using 2D ultrasound (US), where simple manual measurements of anatomical structures are compared against predefined normative charts (Salomon et al. 2019). When central nervous system (CNS) abnormalities are detected, patients are referred for magnetic resonance imaging (MRI) for confirmation or additional evaluation, where available (Prayer et al. 2023).

To compare growth across modalities (e.g., between the initial US scan and any subsequent MRI scan), it is essential to determine whether image‐derived phenotypes (IDPs)—scalar metrics such as volume or diameter of a structure—are comparable between US and MRI. The fundamentally differing physical signals captured by these modalities result in starkly distinct anatomical appearances (Blondiaux and Garel 2013), raising questions about the equivalence of modality‐specific IDPs. Furthermore, as MRI becomes more widely adopted in clinical practice, assessing whether MRI‐derived IDPs align with existing US‐based reference charts is critical to maintaining the best standards of clinical care.

Early studies explored how the two modalities interact with fetal brain tissues, initially focusing on qualitative similarities and differences in structural appearance (Blondiaux and Garel 2013; Pistorius et al. 2008; Malinger et al. 2004; Pooh et al. 2006) before examining discrepancies in standard 2D measurements (Bookstein et al. 2024; Haratz et al. 2010; Matthew et al. 2018; Parkar et al. 2010; Behrendt et al. 2017). While certain IDPs, such as cerebellar diameter (Bookstein et al. 2024), have shown strong agreement, others reveal systematic differences. For instance, lateral ventricle diameters may deviate by over 10% between modalities (Behrendt et al. 2017), potentially leading to conflicting classifications of ventriculomegaly (Perlman et al. 2014). These findings suggest that IDP agreement is structure‐dependent, with potential implications for diagnosis or treatment pathways. Comprehensive investigations of measurement agreement are therefore essential, particularly at the level of individual structures.

To date, studies of modality‐specific IDP agreement have been limited to 2D imaging. However, 2D measurements lack the valuable volumetric detail provided by three‐dimensional (3D) imaging, which is increasingly recognized as superior for diagnosing, monitoring, and understanding pathology (Merz and Pashaj 2017; Pistorius et al. 2008). Advances in fetal MRI and 3D US, combined with automated deep‐learning‐based segmentation, now enable rapid volumetric analysis of brain structures (Wyburd et al. 2020; Uus 2023; Hesse et al. 2022; Venturini et al. 2020), driving their growing adoption in clinical and research settings (Mozaffari and Lee 2017; Paladini et al. 2021). These developments facilitate investigations into 3D structural growth, making it increasingly important to establish the expected agreement IDPs across modalities.

This study investigates the agreement of IDPs from eight brain structures derived from same‐day MRI and 3D US volumes. We focus on the correspondence of volumetric measures during the second trimester, as this is the critical period for anomaly screening (Salomon et al. 2022) and, thus, when MRI or 3D US scan is likely to be used for detailed monitoring. By quantifying the agreement between these modalities, we aim to provide a foundation for integrating 3D neuroimaging into clinical workflows and advancing the understanding of fetal brain development.

## 2 Methods

### 2.1 Data Inclusion and Collection

Pregnant women were recruited from Antenatal Clinics at St Thomas' Hospital, London during their routine clinical US scan, starting at 18^+0days^ gestational weeks (GW). Women were initially given information about the study at their 12‐week scan and then recruited into the study at their routine anomaly scan. Exclusion criteria included: inability to understand the study information sheet, contraindications for MRI scanning (e.g., metal implants), and inability to fit in the scanner bore due to maternal size. The cohort contained a subset of subjects with abnormal findings, provided there were no extreme anatomical deviations (e.g., presence and preserved global shape of all brain regions and absence of lesions). The abnormal findings included subsets with congenital diaphragmatic hernia, cleft lip or palate, and spina bifida. Ethical approval was granted by the London Bridge Research Ethics Committee (ethics No. 14/LO/1806) and informed written consent was obtained from all participants for enrollment in the Intelligent Fetal Imaging and Diagnosis study (iFIND, https://www.ifindproject.com/).

All data were acquired at St. Thomas' Hospital, London. The fetal MRI datasets were obtained on a 1.5 T Philips Ingenia MRI system using a 28‐channel torso coil. Between 9 and 11 single‐shot fast spin echo images were acquired in multiple non‐coplanar geometries with TE = 80/180 ms, TR = 15,000 ms, acquisition resolution 1.25 × 1.25 mm, slice thickness 2.5 mm, and slice spacing 1.25mm gap. The stacks were acquired under different orientations, with 100–160 slices per stack, depending on GA and orientation. Each of the datasets contains 9–11 stacks with a minimum 3 different orientations without major SNR loss.

The fetal US volumes were acquired by one of two qualified obstetric sonographers (JM and ES, who at the time of scanning had > 10 and > 5 years' experience post qualification, respectively), in a dedicated 3D US research clinic, with a Phillips EpiQ US system using an X6‐1 MHz 3D matrix transducer (Philips Healthcare, Best, Netherlands).

The US acquisition protocol included one to three head volume captures per subject, with multiple volumes acquired if the initial image quality was considered suboptimal by the sonographer. The region of interest (ROI) was placed using the axial transventricular standard plane as a reference slice in order to capture the whole brain region with a standardized 3D sweep. A high‐resolution acquisition setting was used by default; however, it was lowered in the presence of excess fetal movement to increase the frame rate and reduce motion artefact. The ROI box was kept as small as possible to maximize spatial resolution and reduce acquisition time by adjusting the ROI width and depth; however, a large sweep angle (slice direction) of 90° was used to capture the complete cranium.

The MRI and US were paired datasets collected on the same day, or within 4 days if same‐day scanning was not practically feasible for the participant. As the US preprocessing pipelines were designed for data collected in the second trimester, only participants scanned between 18^+0days^ and 26^+6days^ GW were included. This is also the period where the anomaly screening is usually performed in routine care and thus a likely time when comparison between US and MRI measures would be made.

### 2.2 MRI Processing

For each subject, all stacks of T2‐weighted (T2w) MRI brain images were reconstructed into a single motion‐corrected 3D volume using fully automated slice‐to‐volume reconstruction (SVR) pipeline, which automatically excludes low quality stacks (Uus et al. 2025; Kuklisova‐Murgasova et al. 2012). The reconstructed voxel size was $0.5^{3}mm^{3}$, and all brain volumes were automatically reoriented to a standard radiological space (Uus et al. 2025). The quality of the SVR‐reconstructed and reoriented images was graded by an experienced neuroscientist (VK), using a 4 grade protocol outlined in (Uus, Egloff Collado, et al. 2023). Only datasets with acceptable to excellent quality and sufficient visibility of all brain structures and anatomical features were included. SVR reconstructions with severe intensity artefacts or failed motion correction were excluded from this study.

The automated fetal brain MRI segmentation pipeline, BOUNTI, was used to segment all 3D SVR brain images (Uus 2023). The BOUNTI segmentation network was trained on TE = 80, 180, and 250 ms datasets, and on normal control and abnormal cases (including ventriculomegaly); see (Uus 2023) for a detailed overview of the training set.

All processing was performed using the auto‐proc‐svrtk toolbox.^1^

**FIGURE 1:**
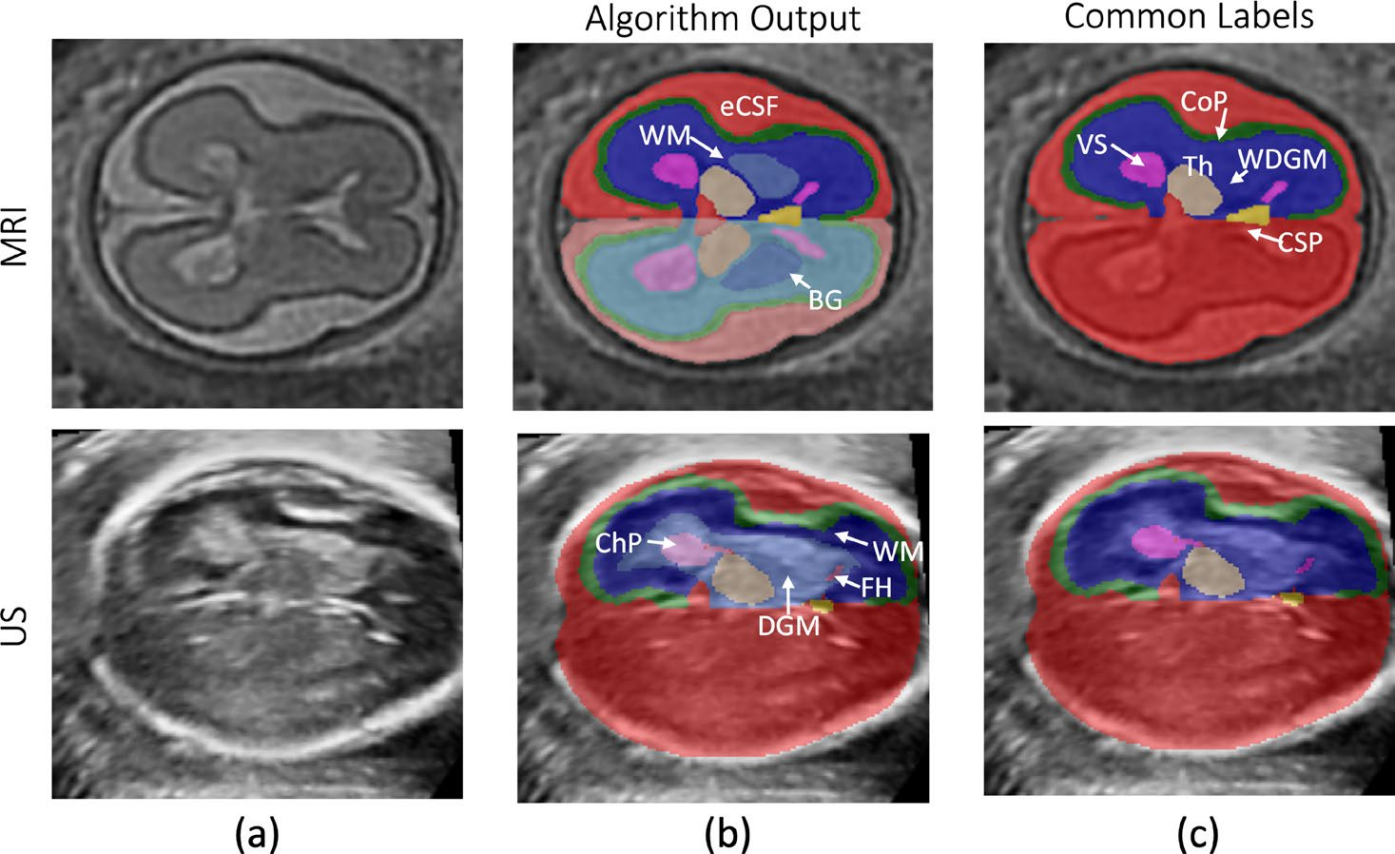
(a) Tissue labelling for a MRI and US scan pair at 25GW. (b) The output from the MRI and US deep‐learning algorithms, with the arrows pointing to structures that differ between the two outputs. (c) The common labels, generated by combining structures from the middle columns. Only the distal hemisphere (furthest from the US probe) was used, as large acoustic artefacts obstruct the anatomy in US on the proximal hemisphere. A full breakdown of combined labels to generate the common labels is shown in Table S1. The arrows are pointing at the following anatomical labels: White matter (WM), extracerebral cerebrospinal fluid (eCSF), basal ganglia (BG), choroid plexus (ChP), frontal horns (FH), deep grey matter (DGM), ventricular system (VS), thalamus (Th), white and deep grey matter (WDGM), cortical plate (CoP) and cavum septum pellucidum (CSP).

The resulting brain segmentations included 19 labels for the major tissue compartments (with left/right separation indicated by *), namely, extracerebral cerebrospinal fluid (eCSF)*, cortical plate (CoP)*, white matter (WM)*, lateral ventricles (VS)*, cavum septum pellucidum (CSP), brainstem (BS), cerebellum (CB)*, cerebellar vermis (CBv), basal ganglia (BG)*, thalamus (Th)*, third ventricle (tV), and fourth ventricle (fV), as shown in Figure 1b. The quality of segmentation was visually graded based on the accuracy of brain tissue interfaces by an experienced neuroscientist.

### 2.3 Ultrasound Processing

Volumetric US inclusion was based solely on image quality, following the protocol outlined in (Namburete et al. 2023). MW and ND manually inspected each US head volume, selecting only those where the skull was fully contained within the 3D US sweep. Each included head volume was resampled to a voxel size of $0.6^{3}\;{\mathrm{mm}}^{3}$ using trilinear interpolation and aligned to a common reference space using the BEAN algorithm (Moser et al. 2022). The volumes were then cropped to $160^{3}$ voxels centered on the brain.

Each aligned volume was segmented into 11 tissue types using two deep‐learning algorithms (Wyburd et al. 2021). First, a U‐Net style model was used to extract the brain, which was then segmented into 10 structures, namely, cortical plate (CoP), cerebellum (CB), CSP, choroid plexus (ChP), posterior horns (PH), frontal horns (FH), white matter (WM), deep grey matter (DGM), BS, and thalamus (Th), using a template‐based deformation model, TEDS‐Net (Wyburd et al. 2021). An example segmentation map is shown in Figure 1b. Due to the strong shadowing artefacts observed in US images on the proximal cerebral hemisphere, only the structures in the distal hemisphere were labelled and the segmentation method returned which hemisphere (left or right) was analyzed. Both algorithms were trained using data collected as part of the INTERGROWTH‐21st Fetal Growth Longitudinal Study (FGLS) (Villar et al. 2014). The quality of each segmentation was manually assessed by ND and MW, following the same grading protocol used for MR image quality assessment.

The US segmentation algorithms were trained exclusively on healthy subjects (Villar et al. 2014), and may therefore be unsuitable for subjects with large anatomical abnormalities. For subjects with ventriculomegaly, manual segmentation of the US images was performed by MKW following the initial US labeling protocol.

### 2.4 Volumetric Measures

Due to the inherent differences in contrast mechanisms between MRI and US, different structures are visible in the volumes obtained from these modalities, as shown in Figure 1a. As such, the available predeveloped processing pipelines delineate different structures (Figure 1b). To facilitate volumetric comparison, a common set of labels was constructed based on the overlap of the available segmentations, as shown in Figure 1c. The included structures were intracranial volume (ICV), CoP, CB, CSP, VS, combined white and deep grey matter (WDGM), BS, and Th.

For MRI, the combined WDGM label was created by merging WM with BG. Similarly, the mid and the distal cerebellum regions were merged to generate the CB label. Since only the distal hemisphere was labeled in the US volumes, we only included MRI labels from the corresponding hemisphere for each subject individually to ensure the same hemisphere was measured between modalities. For US, the VS label was generated by combining the ChP, PH, and FH. Additionally, the WM and DGM labels were combined to produce WDGM. A detailed breakdown of label combinations is provided in Table S1.

For each scan, the volume of each label was computed by summing the number of voxels assigned to that structure before multiplying by the voxel spacing.

### 2.5 Statistical Analysis

The differences between IDPs derived from US and MRI volumes for each structure were quantified using two metrics: the mean absolute difference (MAD) and the mean absolute relative difference (MARD). MAD expresses the average difference between the two modalities in cubic centimetres ($cm^{3}$), while MARD reports the differences as a percentage of the average structure size. Mathematically, they can be written as:

$$\mathrm{MAD}=\frac{1}{N}\sum_{i=1}^{N}\left|v_{i}^{\mathrm{US}}-v_{i}^{\mathrm{MRI}}\right|$$

$$\text{MARD}=\frac{1}{N}\sum_{i=1}^{N}\frac{\left|v_{i}^{\mathrm{US}}-v_{i}^{\mathrm{MRI}}\right|}{v_{i}^{\mathrm{AVG}}}\times 100$$

where $v_{i}^{\mathrm{US}}$ is the US‐derived volume, $v_{i}^{\mathrm{MRI}}$ the corresponding MRI‐derived volume for subject i, and $v_{i}^{\mathrm{AVG}}$ is the average of the two volumes:

$$v_{i}^{\mathrm{AVG}}=\left(v_{i}^{\mathrm{US}}+v_{i}^{\mathrm{MRI}}\right)/2$$

here, N represents the number of subjects included in the analysis.

To evaluate the degree of agreement between volume measurements for each subject, we used the two‐way random‐effect model with a single‐rater interclass correlation coefficient $\left(\mathrm{ICC}\right)$ (Shrout and Fleiss 1979). Agreement was classified as poor (ICC<0.40), moderate (0.40 < *ICC* < 0.60), good (0.60<ICC<0.75), or excellent (ICC>0.75) (Koo and Li 2016). We also report the 95% confidence intervals for ICC values, to reflect the range of agreement across the subjects. ICC calculations were performed using the *pingouin* Python package (version 0.5.4) (Vallat 2018).

To estimate the correlation between the US and MRI same‐day volume measurements, the Pearson correlation coefficient $\left(r\right)$ and a paired sampled *t*‐test $\left(p\right)$ were computed. A line of best fit to describe the relationship between the US‐ and MRI‐derived measurements was calculated using linear regression. The slope, intercept, and standard error $\left(\mathrm{SE}\right)$ of the fit are reported.

## 3 Results

### 3.1 Subject Inclusion

**FIGURE 2:**
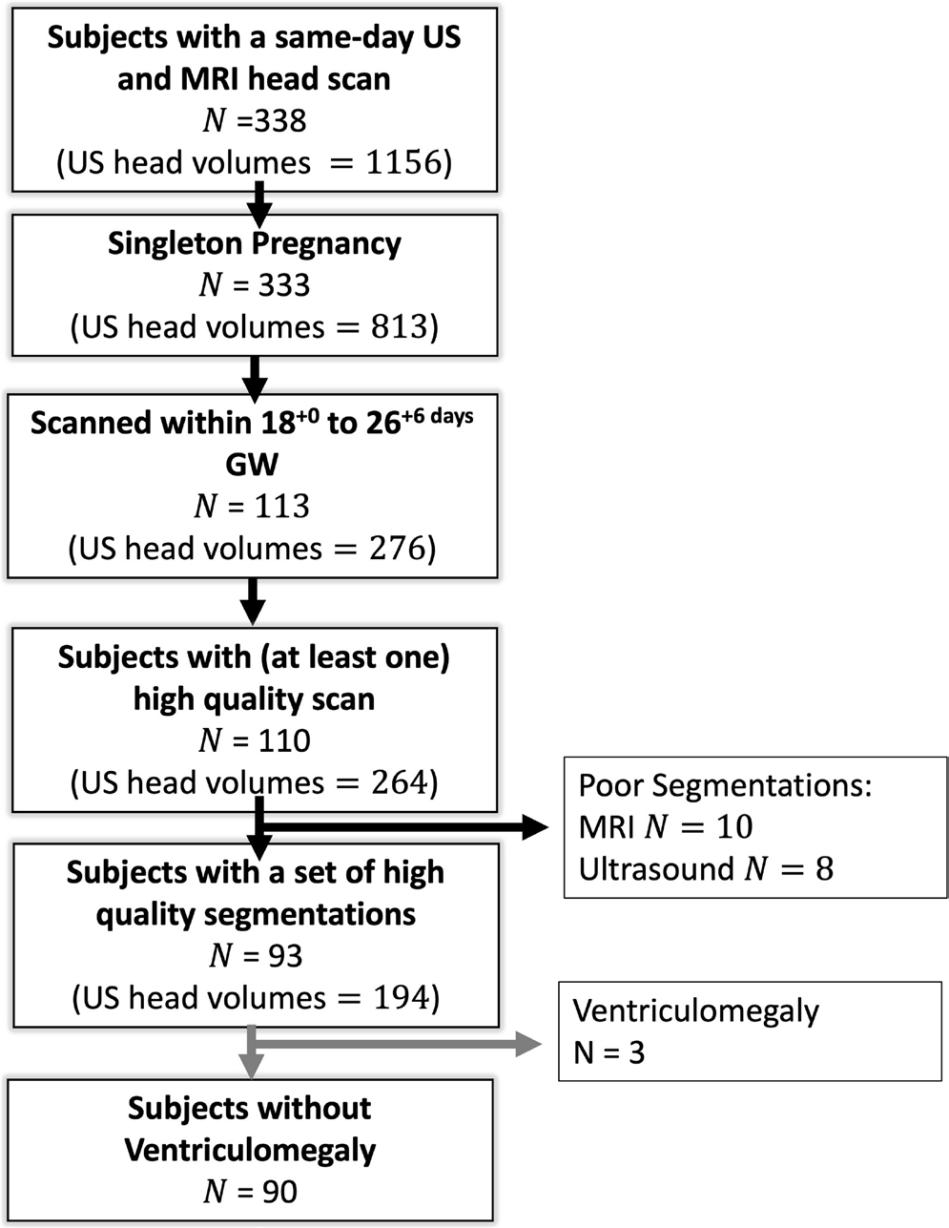
Flow diagram of subject inclusion in the study, where N is the number of subjects. As multiple US head volumes were usually acquired for each subject (volumes per subject: Median 2, mode 2 and a maximum of 7), we have also included the number of US volumes at each stage of the pipeline.

A flowchart providing an overview of subject inclusion is presented in Figure 2. A total of 338 women underwent same‐day MRI and US head volume scans. Of these, five were excluded due to multiple pregnancies, as it was not possible to distinguish between fetuses across modalities. The cohort was then filtered by gestational age at scan, with 113 participants being scanned between 18^+0days^ and 26^+6days^ GW, the gestational age range of interest. Three additional participants were excluded due to poor‐quality US volumes.

**FIGURE 3:**
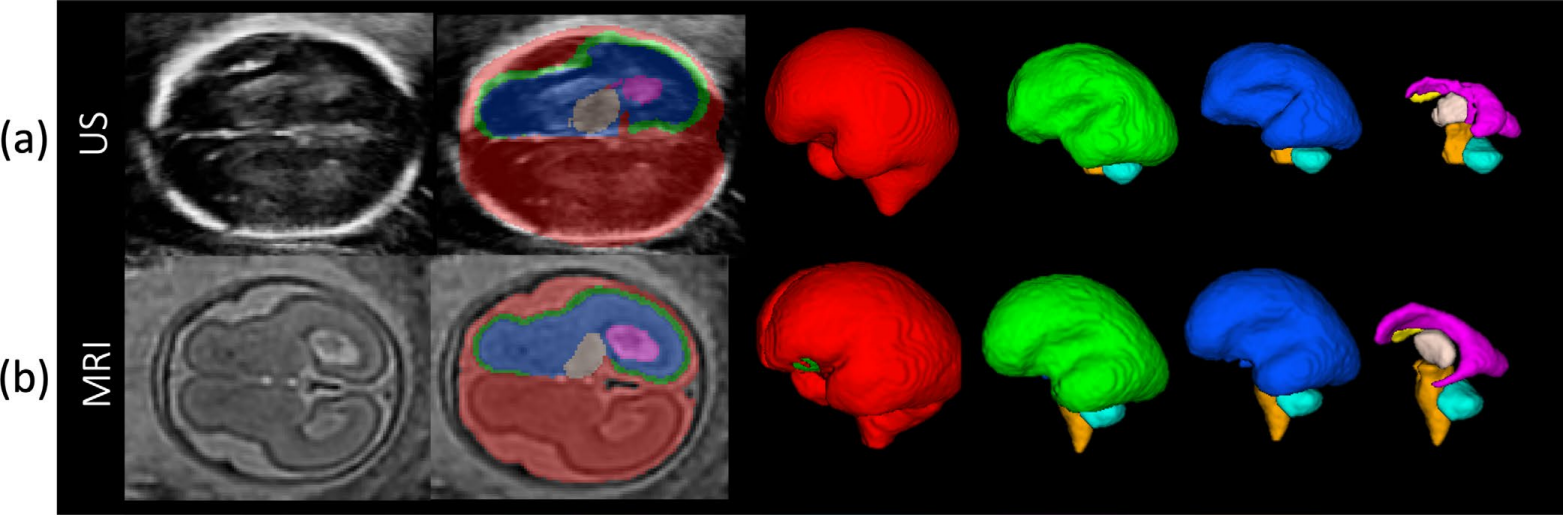
An overview of the segmentations from (a) US and (b) MRI. The ICV is shown in red, CoP in green, WDGM in blue, ventricular system in pink, Th in beige, CSP in yellow, CB in cyan, and BS in orange. Tissues are only segmented in the distal hemisphere (the hemisphere furthest from the probe), except ICV which is extracted across both hemispheres.

Following quality assessment, 10 subjects were excluded for poor MRI segmentation quality, and eight for poor US segmentation quality, with one subject excluded due to both modalities. This resulted in 93 subjects with high‐quality segmentations from *same‐day* MRI and US scans. Examples of included segmentations are shown in Figure 3, with segmentation grades for each modality provided in Figure S1. From this cohort, we excluded any subject with ventriculomegaly from the main analysis due to large anatomical anomalies but analyzed them separately, resulting in a final cohort of 90 subjects.

It is worth noting that, for each subject, only a single MRI head volume was reconstructed and analyzed during the scanning session. In contrast, during the US scanning session, multiple head volumes were often acquired and later analyzed (head volumes per subject: mean = 2.4, median = 2, mode = 2, maximum = 7). The US segmentation pipeline was applied to all available US head volumes (264 volumes), with 46 volumes excluded due to poor segmentation quality.

The majority of these subjects (53/90) had the US and MRI scans performed on the same day, with the largest interval between scans being 4 days (mean difference: 1.2 days, median difference: 0 days, mode difference: 0 difference, maximum difference: 4 days).

### 3.2 Volumetric Comparisons

**FIGURE 4:**
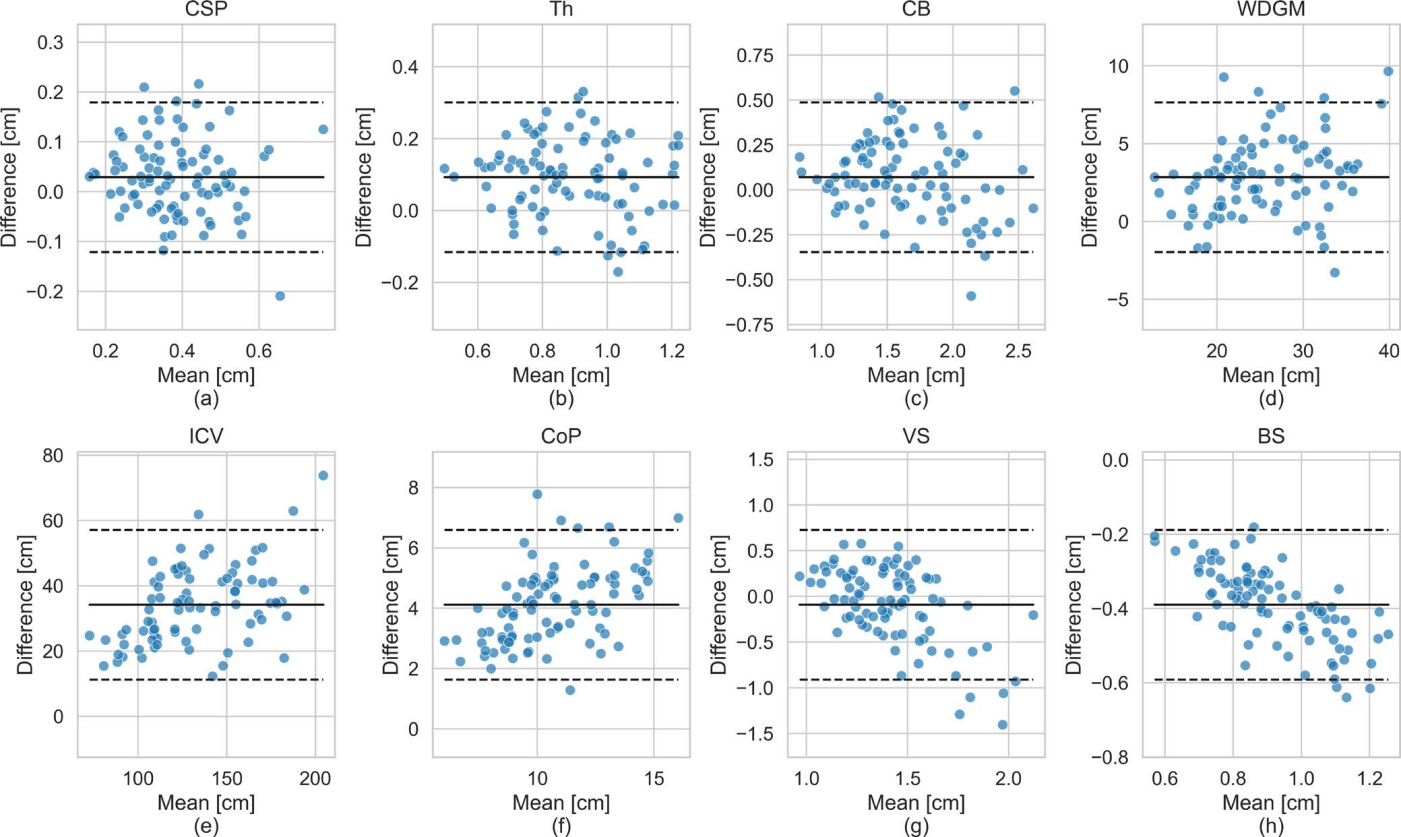
Bland–Altman plots describing the agreement between same‐day US and MRI volume measurements. The solid line indicates the mean difference between the modalities for each measure ($v_{i}^{\mathrm{US}}$ – $v_{i}^{\mathrm{MRI}}$). The dotted lines represent ±1.96 standard deviations (SD) from the mean. The difference was calculated by subtracting the MRI volume from the US volume, and thus if the mean is positive the US had an average greater volume for that structure.

**FIGURE 5:**
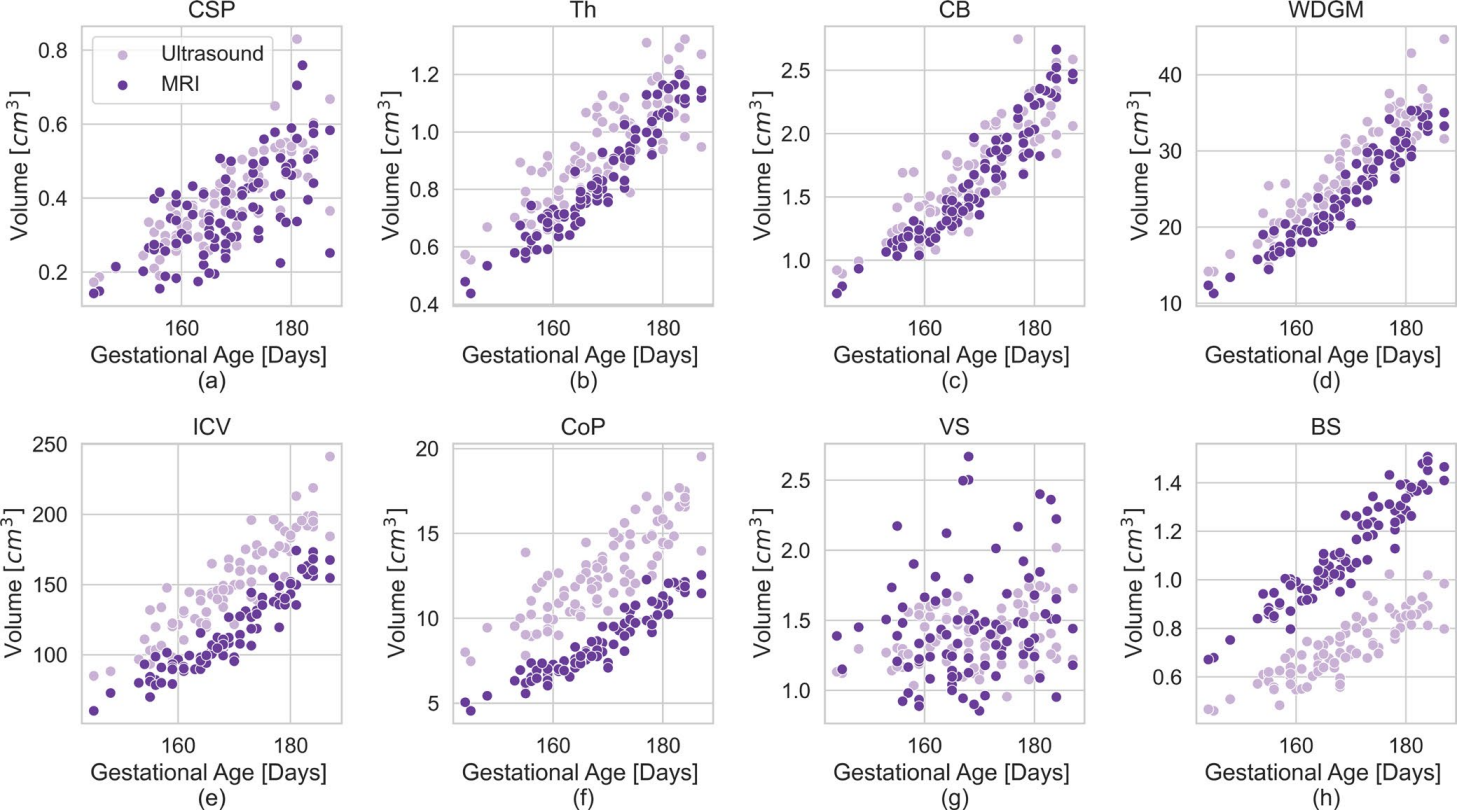
IDP for each structure over gestational age. The different shades represent which modality, MRI or US, the IDP was measured from.

**FIGURE 6:**
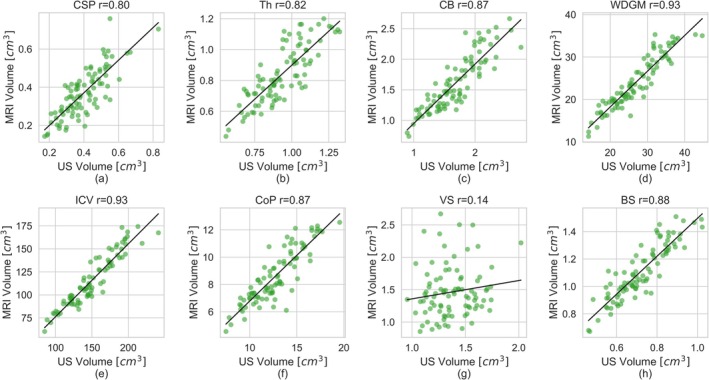
The MR‐derived IDP plotted against the US‐derived IDP of the same subject to show the agreement over size. Each data point is a unique subject. A line of best fit describing the correlation is included.

Figure 4 presents Bland–Altman plots for all IDPs across subjects, while Figure 5 shows the corresponding scatter plots, and Figure 6 shows the correlation between the MRI‐ and US‐derived IDPs.

#### 3.2.1 Cavum Septum Pellucidum, Thalamus, Cerebellum and WDGM

The IDPs for the CSP, Th, and CB demonstrated high consistency between the two modalities for each subject, achieving excellent or good agreement $\left(\mathrm{ICC}>0.72\right)$ and low mean absolute difference relative to volume $\left(\text{MARD}<14\%\right)$, as detailed in Table 1. The Bland–Altman plots (Figure 4a–d) further support this, showing mean volume differences close to zero for these structures. Further, the random distribution of data points around the mean in the Bland–Altman suggests that the relationship between modalities is consistent with mean volume. This strong agreement is corroborated by the scatter points (Figure 5a–d) and correlation plots (Figure 6a–d), where data points show a high degree of overlap and a strong positive correlation $\left(r>0.79\right)$, as summarized in Table 2.

**TABLE 1:** An evaluation of the agreement in volume measurements between the two modalities. Excellent ICC agreement (ICC>0.75) is indicated with ** and good agreement (0.60<ICC<0.75) with *. Note that the MAD is the absolute of the mean differences shown in Figure 4. The arrows indicate whether a lower (↓) or higher (↑) value corresponds to a better agreement.

Structure	MAD (cm^3^) (↓)	MARD (%) (↓)	ICC (↑)	95% CI	*p*
ICV	34.5 ± 12.0	26 ± 8	0.56	[−0.05, 0.86]	<0.001
Cortical Plate (CoP)	4.14 ± 1.20	39 ± 11	0.32	[−0.04, 0.69]	<0.001
WDGM	3.16 ± 2.21	12 ± 8	0.84**	[0.19, 0.94]	<0.001
Cavum Septum (CSP)	0.07 ± 0.06	18 ± 15	0.77**	[0.64, 0.85]	<0.001
Cerebellum (CB)	0.18 ± 0.14	11 ± 8	0.86**	[0.79, 0.91]	<0.001
Ventricular System (VS)	0.36 ± 0.37	23 ± 18	0.13	[−0.07, 0.32]	0.1
Thalamus (Th)	0.12 ± 0.08	14 ± 9	0.72*	[0.23, 0.88]	<0.001
Brainstem (BS)	0.39 ± 0.10	42 ± 8	0.22	[−0.03, 0.58]	<0.001

**TABLE 2:** Correlation between the volume measurements computed from same‐day US and MRI volumes. Pearson correlation coefficient $\left(r\right)$ and a two‐sided paired *t*‐tes*t* $\left(p\right)$ was performed for each structure, to investigate if there was a correlation between the modalities and whether it was statistically significant.

	Slope	Intercept	*r*	*p*	SE
ICV	0.783	−18.2	0.94	<0.001	0.031
Cortical plate (CoP)	0.666	1.23	0.88	<0.001	0.038
WDGM	0.831	16.4	0.93	<0.001	0.035
Cavum septum (CSP)	0.867	0.23	0.79	<0.001	0.07
Cerebellum (CB)	0.969	−0.19	0.88	<0.001	0.055
Ventricular System (VS)	0.458	8.77	0.19	0.069	0.249
Thalamus (Th)	0.871	2.49	0.82	<0.001	0.063
Brainstem (BS)	1.345	1.37	0.88	<0.001	0.076

Similarly, the derived measures of WDGM volume also showed excellent agreement between the modalities (intra‐class correlation, ICC=0.84) and a strong positive correlation $\left(r=0.93,\;p<0.001\right)$. However, there was a slight bias toward larger US‐derived IDPs compared to MRI, with a mean difference of 3.16 cm^3^ (12% of the mean volume).

#### 3.2.2 Intracranial and Cortical Plate Volume

The volumes of ICV and CoP followed similar trajectories across gestational age in both modalities, as shown in Figure 5e,f. The measurements were highly correlated ($r_{\mathrm{ICV}}=0.94,p_{\mathrm{ICV}}<0.001,r_{\mathrm{CoP}}=0.88,p_{\mathrm{CoP}}\;<0.001$). However, for both structures, the US‐derived IDPs were systematically larger than MRI IDPs, as shown in Figures 4e,f and 5e,f.

For ICV, the mean difference between modalities was 35.5 cm^3^, representing 26% of the mean volume, resulting in a moderate agreement (ICC=0.56). Conversely, for CoP, the mean difference was 4.14 cm^3^, accounting for 39% of the mean volume. This substantial discrepancy, relative to the overall size of the structure, was reflected in the poor agreement score (ICC=0.32).

#### 3.2.3 Ventricular System

The VS appears notably different between MRI and US, leading to the development of separate segmentation protocols for each modality. In the US pipeline, lateral ventricle regions (i.e., PH, ChP, and FH) were grouped under the umbrella term “ventricular system”, whereas the MRI pipeline segmented the VS as a single unit. Combining separately structures segmented often leads to gaps within the merged structure, as shown in Figure 3. These differences in segmentation protocols likely contributed to the poor agreement between modalities $\left(\mathrm{ICC}=0.127\right)$. Furthermore, the ventricular volumes showed no significant correlation between modalities $\left(r=0.07,p<0.19\right)$, suggesting subject‐level discrepancies rather than a systematic shift.

**FIGURE 7:**
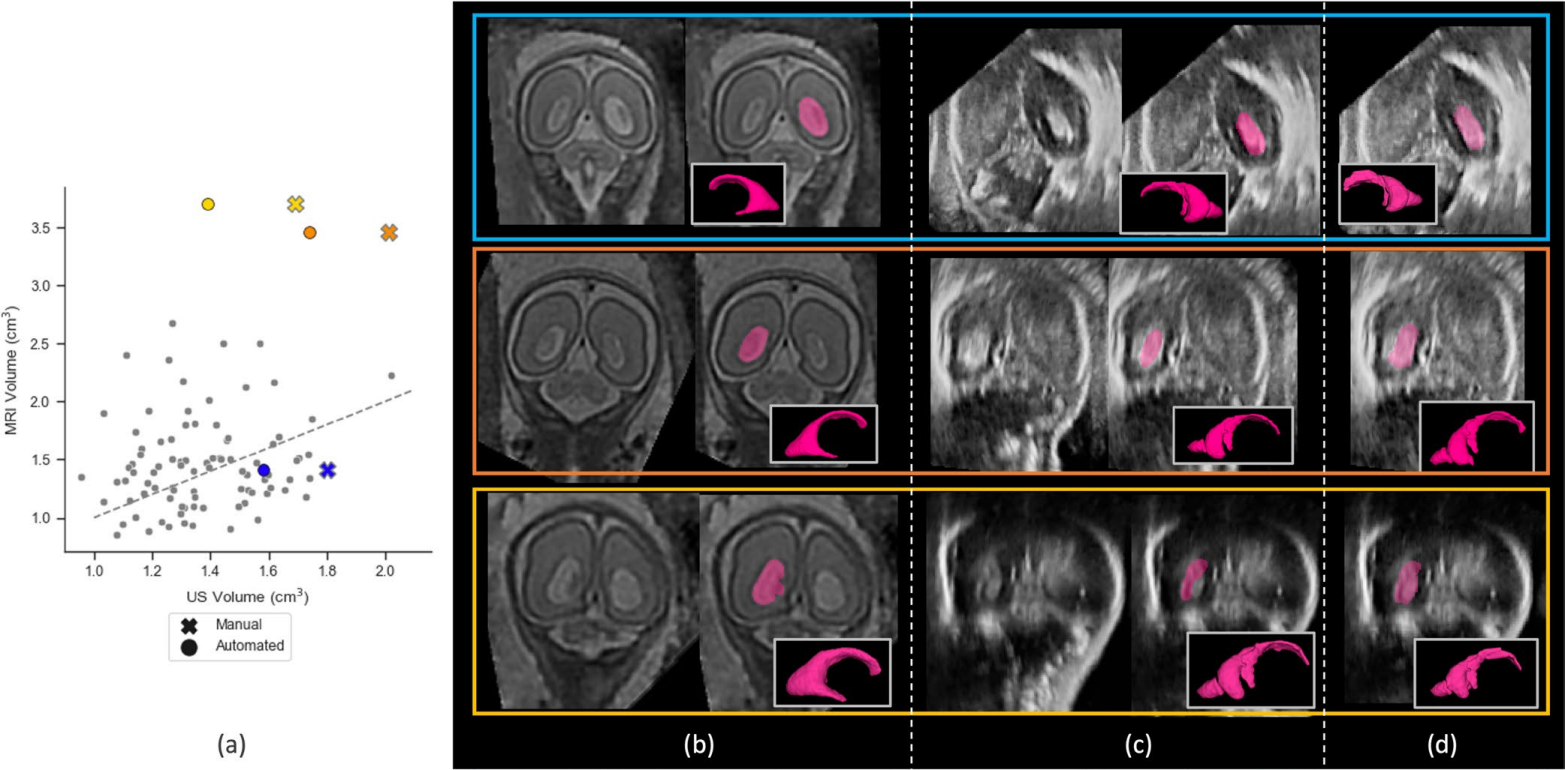
An overview of the subjects with ventriculomegaly. The blue and orange subjects had mild ventriculomegaly whereas the orange was severe. (a) the correlation plots with the ventriculomegaly subjects highlighted. The grey dotted line on the LHS is the equality line. The volume computed from the automated deep‐learning algorithms (c) is indicated with an **o** and the volume's from manual segmentations (d) are shown with an **x**. (b) the automated labels computed from the MRI volumes and (c) the US automated labels. (d) The manual VS segmentation from US.

Within the cohort with high‐quality scans, three subjects had ventriculomegaly, two mild and one severe, as assessed by measuring the atrial diameter on US and MRI: mild 10–11.9 mm, moderate 12–14.9 mm, and severe > = 15 mm. Two of these, one mild and one severe, exhibited large discrepancies in VS volume between MRI and US, as highlighted in yellow and orange in Figure 7. In these examples, the US segmentation poorly delineated the VS, whereas the MRI segmentation closely followed the VS's boundaries. Since the US segmentation algorithm was trained exclusively on healthy examples (Villar et al. 2014), it had not encountered such extreme deviations in VS appearance during training. As a result, these examples were out‐of‐distribution (OOD) for the network, leading to poor segmentation performance. Manual segmentation was also performed for these cases, as shown in Figure 7d. In all three instances, manual segmentation yielded larger VS volumes than the deep‐learning model‐derived labels. However, for the two severe cases, the manual US volumes remained much smaller than the MRI‐derived IDPs. MRI is the preferred modality for ventriculomegaly detection (Cardoen et al. 2011), as it provides superior delineation of enlarged ventricles, which can be harder to detect in US (Blondiaux and Garel 2013). This may explain the substantial differences in measurements between the two modalities.

#### 3.2.4 Brainstem

The BS exhibited the largest difference in absolute volume measurements between the two modalities. Brainstem volumes were consistently higher when measured in MRI compared to US, as shown in Figures 4h and 5h. On average, the MRI‐derived volumes were 0.39 cm^3^ greater than those from US, representing 42% of the total BS volume. Moreover, the volume difference increased with the mean volume—and, by extension, gestational age—as evidenced by the negative trend in the Bland–Altman plot points in Figure 4h.

As the second trimester is a period of rapid growth, the volume of each of the eight structures more than doubles across the gestational range studied here. Therefore, it is expected that the differences in volume measurements would scale proportionally to the structure's size. To investigate this relationship further, Figure S2 shows the volume differences as a percentage of the mean size. When normalized by mean volume, the scatter becomes randomly distributed for the BS, suggesting that the observed differences are proportional to the structure's size rather than indicative of systematic bias.

## 4 Discussion

This study compared IDPs of eight brain structures from same‐day fetal MRI and US scans. The cohort consisted of both healthy and atypically developing subjects, but without severe anatomical variations. A key strength of this work is the short interval between cross‐modality scans (maximum of 4‐days), ensuring that observed differences are due to modality‐specific factors rather than fetal growth between scanning sessions. While this allows a robust comparison of relative sizes, the absence of ground truth precludes definitive conclusions about the absolute precision or accuracy of either modality.

### 4.1 Tissue‐Dependent Agreement

The IDP agreement between MRI and US varied across brain structures. Strong agreement was observed for the CSP, CB, and Th, likely due to their clear tissue boundaries in both modalities (Pistorius et al. 2008; Garel and Alberti 2006). These boundaries, enhanced by their central location in the brain and high contrast with surrounding tissues, are less affected by the characteristic US skull shadowing and provide essential image features (e.g., strong edges) for deep‐learning‐based segmentation models (Adebayo et al. 2018). Similar agreement has previously been reported for 2D cerebellar diameter measurements in US and MRI (Bookstein et al. 2024), supporting our findings on the consistency of cerebellar size across modalities.

**FIGURE 8:**
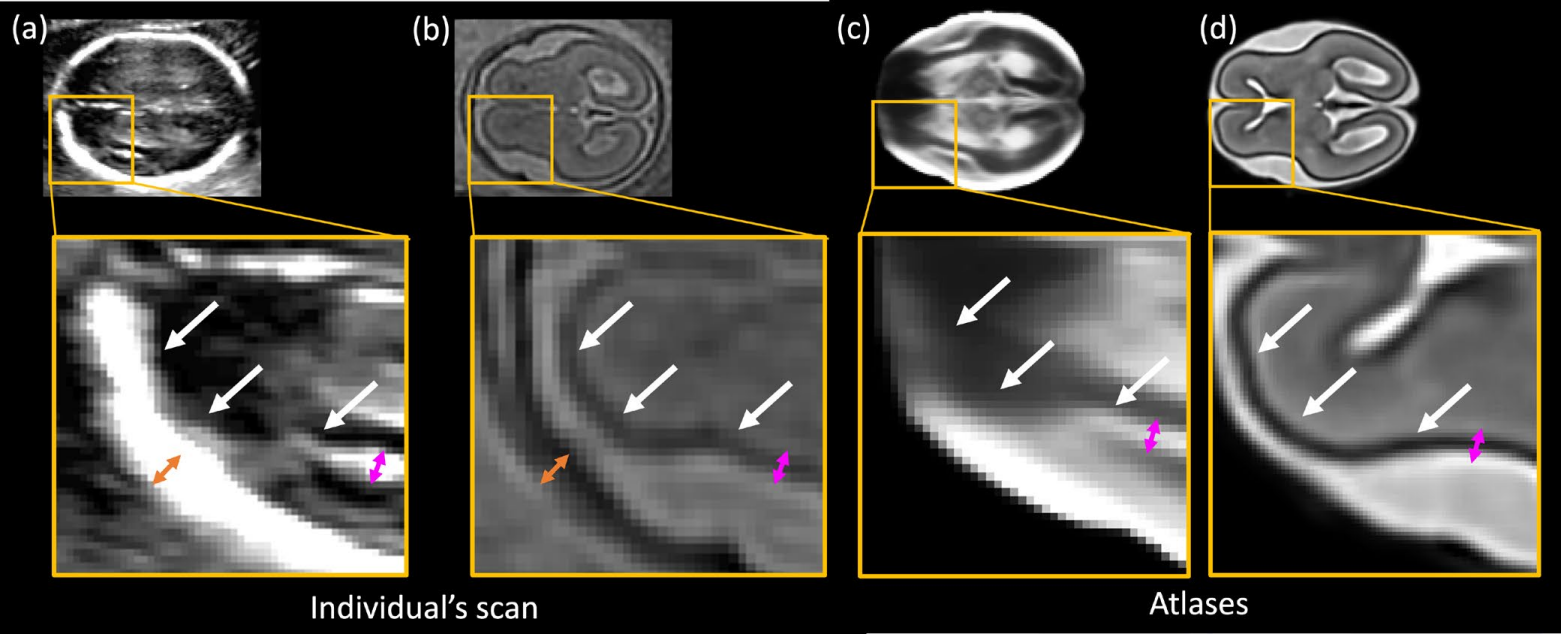
Same‐day US (a) and MRI volume (b) at 21 GW and the atlas volumes from US (c) and MRI (d) at the same age (Namburete et al. 2023; Uus, Kyriakopoulou, et al. 2023). The same size arrows in the same position have been added to each. The white arrows show the cortical plate boundary, the orange indicates the skull thickness based on the MRI, showing the US skull appears thicker and the pink arrows show the CoP ribbon, modelled on the US volume. In the US volume, regions of the pericerebral space, that are visible in the MRI, are missing. This study investigates the agreement of image‐derived phenotypes (IDPs) from eight fetal brain structures derived from same‐day MRI and 3D US volumes. Strong agreement was observed for the CSP, Th, CB, WMDGM, whereas systematic biases were revealed for ICV and CoP.

Excellent agreement was also found for WDGM volumes, as both tissues exhibit strong contrast with surrounding tissues in MRI and are well‐defined in US, except in the posterior and anterior regions of the brain that are strongly affected by skull shadowing (Figure 8a). The selected segmentation pipeline for US mitigated this issue by guiding anatomical delineation in areas with artefacts, enabling accurate white matter segmentation (Wyburd et al. 2021).

In contrast, cortical plate (CoP) and ICV IDPs were systematically larger in US compared to MRI. In fetal MRI, there is clear separation between the cortex, skull, and pericerebral space, which allows precise delineation of these structures (Figure 8b,d). However, in US, heavy artefacts caused by the high acoustic impedance of the skull often obstruct the pericerebral space, making it difficult to distinguish between the cortex and the skull, particularly in the anterior and posterior brain regions (Figure 8a,c). This ambiguity in the tissue boundaries is illustrated by the white arrows in Figure 8a, where the cortex appears to directly neighbor the skull. It is, therefore, not always possible to disentangle the boundaries of these structures in US volumes. These artifacts likely contribute to the systematic differences observed in both CoP and ICV volumes, with US‐derived IDPs consistently larger than their paired MRI‐derived values. The cortical ribbon and skull both appear notably thicker in US than in MRI, as indicated by the pink and orange arrows in Figure 8, further amplifying these volume discrepancies.

The ventricular system (VS) presented the poorest agreement between modalities, with a lack of correlation between the IDPs and notable discrepancies in segmentation. These differences stem from the vastly different appearance of the VS in MRI and US, as shown in Figure 3. In MRI, ventricles are well‐defined due to the strong contrast between cerebrospinal fluid and surrounding tissues, while in US, the ventricular boundaries are often less distinct. Previous studies have reported similar discrepancies in 2D ventricular measures (Behrendt et al. 2017; Garel and Alberti 2006). One study found that ventricles appeared significantly larger (over 10%) in fetal MRI compared to US (Behrendt et al. 2017). Conversely, another study observed a size‐dependent variation: US‐derived ventricular diameters exceeded MRI‐derived ones when below 10 mm, but were smaller when MRI‐derived diameters were greater than 10 mm (Garel and Alberti 2006). These results highlight the challenges in consistently assessing ventricular size across modalities and emphasize the difficulty of developing reliable segmentation methods for this structure.

Brainstem volume comparisons were similarly challenging due to differences in the field of view (FoV) between MRI and US. MRI consistently included a larger FoV, resulting in a larger BS volume, as shown in Figure 5h. These discrepancies are expected to vary further with gestational age, fetal growth, and as the field of view necessarily changes.

### 4.2 Methodological and Modality Considerations

Both MRI and US have inherent limitations for volumetric analysis. US suffers from poor tissue contrast and artefacts, particularly near the skull, which complicates delineation of certain structures and lowers the certainty of the produced segmentations and the precision of computed volumes. Conversely, MRI often has a lower spatial resolution than US, due to large slice thickness, which introduces partial volume effects, where a single voxel may encompass multiple tissue types, leading to volume aberrations (Kurmis et al. 2004). These limitations in both modalities can therefore result in volumetric inaccuracies.

This study focused on the second trimester (between 18^+0days^ and 26^+6days^ GW), the gestational window for routine anomaly screening. It should be noted that the relationship between the two modalities' IDP measurements is likely to differ in the first and third trimesters. In the first trimester, MRI is more susceptible to strong artefacts from fetal motion, while in the third trimester, increased ossification exacerbates shadowing in US, thereby obstructing key anatomical boundaries for segmentation.

The cohort studied here has both typically and atypically developing subjects. However, subjects with large anatomical variation, e.g., ventriculomegaly, were excluded due to the ultrasound segmentation protocol only being designed for optimally developing subjects, and thus unsuitable. Therefore, this comparison between MRI and US‐derived measures may not be valid for atypical cohorts with large anatomical variations.

The use of two deep‐learning algorithms for segmentation was an important aspect in the design of this study. Both algorithms enable near‐instantaneous tissue segmentation, offering a practical and scalable alternative to labor‐intensive and time‐consuming manual labeling, especially for analyzing large cohorts or multiple structures. As deep‐learning methods become increasingly adopted in fetal brain studies, their application here reflects current research practices. Although segmentation outputs may not always be directly comparable—such as for the ventricular system—these algorithms segment what is visible in each modality, making this comparison both relevant and valuable for the research community.

## 5 Conclusion

As fetal MRI and 3D US imaging become increasingly common in fetal analysis, it is important to determine how IDPs differ across these modalities. In this study, we used deep‐learning pipelines to segment the fetal brain of a cohort with no severe anatomical deviation and compared the derived volumes of key structures from same‐day MRI and US scans. Volumes of the CSP, Th, CB, and WDGM were found to be comparable between the two modalities. However, ICV and CoP showed strong correlations but with systematic shifts in absolute volume. In contrast, the VS and BS were not comparable, likely due to inconsistent delineation between the segmentation methods. These structure‐specific findings highlight the need for careful consideration to ensure reliable comparisons between these imaging modalities in both clinical and research settings.

## Supporting information


**Figure S1:** Examples of each grade type from both MRI and US. Grade 2, shows the highest quality scans, grade 1, the intermediate scans and grade 0 the poor scans which were excluded. Example (e) shows a subject with severe ventriculomegaly.

**Figure S2:** land–Altman plots describing the agreement between same‐day US and MRI volume measurements, with the difference shown as a percentage of mean size. The difference was calculated by subtracting the MRI volume from the US volume before dividing by the average of the two, and thus if the mean is positive, it means the US had an average greater volume.

**Table S1:** An overview of how the common space labels were created.

## Data Availability

Research data are not shared.

## Endnotes

^1^

https://github.com/SVRTK/SVRTK.
