# Supplementary figures and images for: Cross‐Modality Comparison of Fetal Brain Phenotypes: Insights From Short‐Interval Second‐Trimester MRI and Ultrasound Imaging
Source: Hum Brain Mapp. 2025 Oct 1;46(14):e70349. doi: 10.1002/hbm.70349 (PMC12485670; doi:10.1002/hbm.70349)

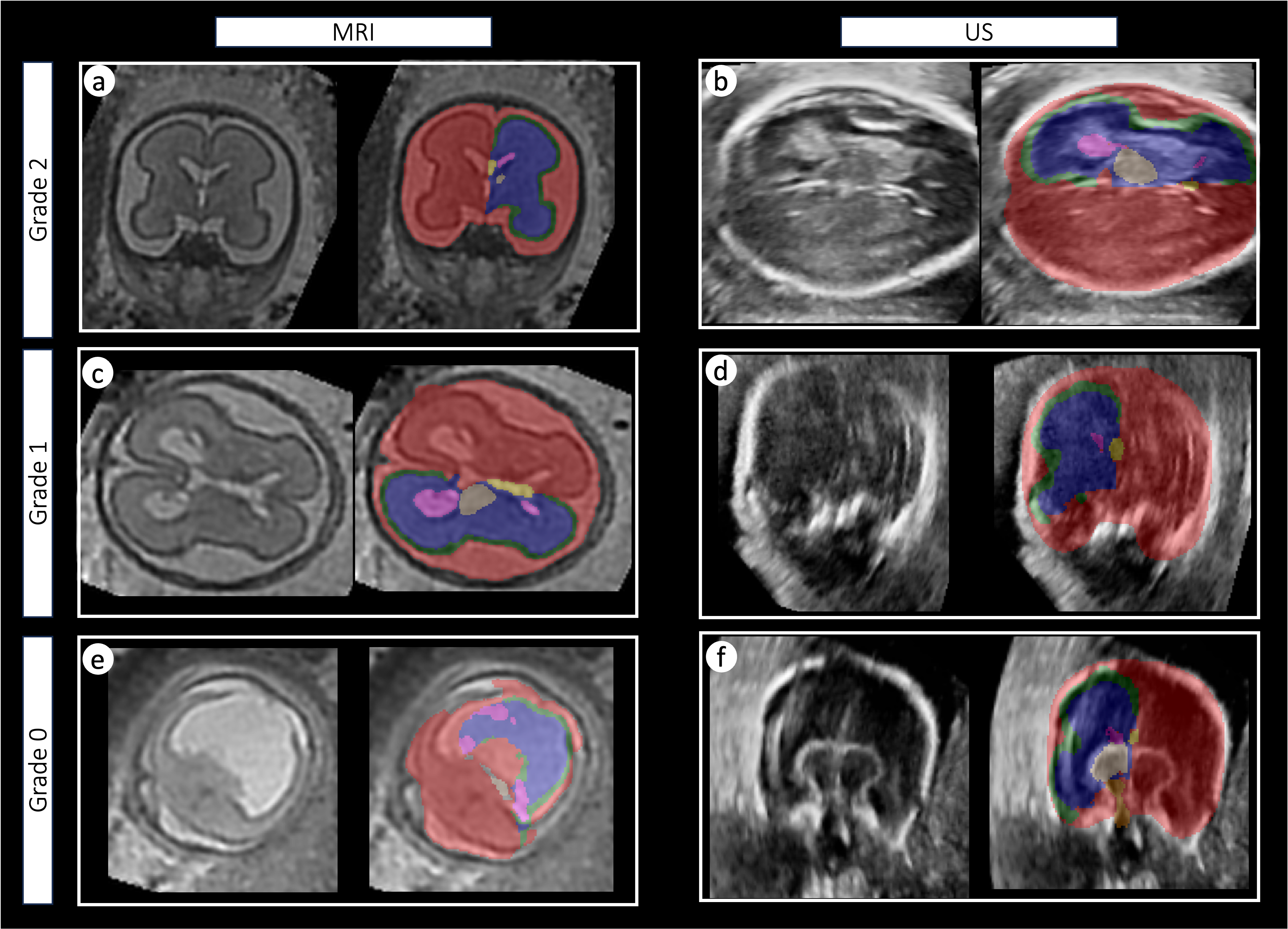

Supplement: Supplementary file 1 — Figure S1: Examples of each grade type from both MRI and US. Grade 2, shows the highest quality scans, grade 1, the intermediate scans and grade 0 the poor scans which were excluded. Example (e) shows a subject with severe ventriculomegaly. [file HBM-46-e70349-s001.png]

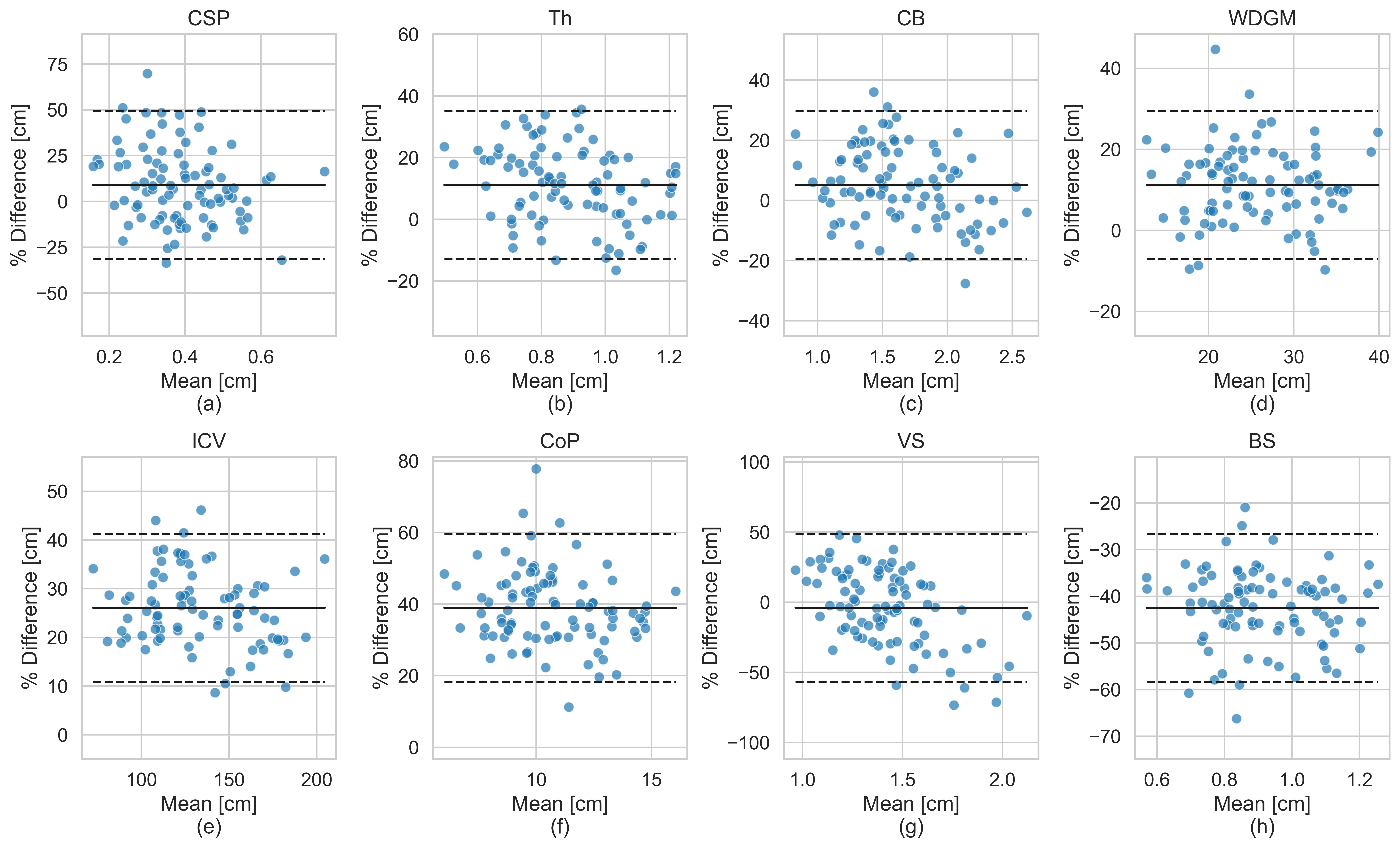

Supplement: Supplementary file 2 — Figure S2: land–Altman plots describing the agreement between same‐day US and MRI volume measurements, with the difference shown as a percentage of mean size. The difference was calculated by subtracting the MRI volume from the US volume before dividing by the average of the two, and thus if the mean is positive, it means the US had an average greater volume. [file HBM-46-e70349-s003.png]
