# Supplementary material for: Cross‐Modality Comparison of Fetal Brain Phenotypes: Insights From Short‐Interval Second‐Trimester MRI and Ultrasound Imaging
Source: Hum Brain Mapp. 2025 Oct 1;46(14):e70349. doi: 10.1002/hbm.70349 (PMC12485670; doi:10.1002/hbm.70349)
Supplement: Supplementary file 3 — Table S1: An overview of how the common space labels were created. [file HBM-46-e70349-s002.docx]

| **Common Label** | **MRI Labels** | **US Labels** |
| --- | --- | --- |
| Inter-cranial volume (ICV) | All labels combined (both hemispheres) | ICV (both hemispheres) |
| Cortical Plate (CoP) | CoP (distal) | CoP (distal) |
| White and deep gray matter (WDGM) | WM (distal) + Pu | WM (distal)+ DGM (distal) |
| Cavum septum pellucidum (CSP) | CSP (distal) | CSP |
| Cerebellum (CB) | CB (distal) + CBV | CB (distal) |
| Ventricular system (VS) | VS (distal) | Parietal horns (PH) (distal)+ Frontal horns (FH) (distal) + Choroid Plexus (ChP) (distal) |
| Thalamus (Th) | Th (distal) | Th (distal) |
| Brainstem (BS) | BS | BS |
